# Some Famous Old Maids

**Published:** 1885-07

**Authors:** 


### Some Famous Old Maids.

Elizabeth of England was one of the most illustrious of modern sovereigns. Her rule over Great Britain certainly comprised the most brilliant literary age of the English-speaking people. Her political acumen was certainly put to as severe tests as that of any other ruler the world ever saw. Maria Edgeworth was an old maid. It was this woman's writings that first suggested the thought of writing similarly to Sir Walter Scott. Her brain might well be called the mother of the Waverly novels. Jane Porter lived and died an old maid. The children of her busy brain were "Thaddeus of Warsaw" and "The Scottish Chiefs," which have moved the hearts of millions with excitement and tears. Joanna Baillie, poet and play-writer, was "one of 'em." Florence Nightingale, most gracious lady, heroine of Inkermann and Balaklava hospitals, has to the present written "Miss" before her name. The man who should marry her might well crave to take the name of Nightingale. Sister Dora, the brave spirit of English pest-houses, whose story is as a helpful evangel, was the bride of the world's sorrow only. And then what names could the writer and reader add of those whom the great world may not know but we know, and the little world of the village, the church, the family know and prize beyond all worlds!

